# Individual, family, and environmental correlates of fundamental motor skills among school-aged children: a cross-sectional study in China

**DOI:** 10.1186/s12889-024-17728-2

**Published:** 2024-01-17

**Authors:** Yuxiu He, Lin Zhou, Wei Liang, Qi Liu, Wanxin Liu, Shijian Wang

**Affiliations:** 1 School of Physical Education, Hebei Normal University, Shijiazhuang, China; 2 Key Laboratory of Measurement and Evaluation in Exercise Bioinformation of Hebei Province, Shijiazhuang, China; 3 College of Physical Education, Shenzhen University, Shenzhen, China

**Keywords:** Fundamental motor skill, Socio-ecological model, Individual factors, Family factors, Environmental factors

## Abstract

**Objective:**

This cross-sectional study examined the socio-ecological factors influencing fundamental motor skills (FMS) in Chinese school-aged children.

**Methods:**

A total of 1012 parent-child pairs were randomly sampled between March-1st and April-15th, 2022. Based on the socio-ecological model of Children’s FMS, three levels of factors: individual-level (e.g., demographic, physical, psychological, and behavioral characteristics of children), family-level (e.g., caregiver demographics, parental support, and socioeconomic status), and environmental factors (e.g., availability of physical activity equipment) were assessed using self-reported scales (e.g., the Self-perception Profile for Children, the Physical Activity Enjoyment Scale, and the 12-item Psychological Well-Being Scale for Children) and objective measures (e.g., ActiGraph GT3X, the Chinese National Student Physical Fitness Standard, and the Test of Gross Motor Development-Third Edition). Multi-level regression models were employed using SPSS.

**Results:**

The results demonstrated that children’s age, sex, physical fitness, parental support, and the quality of home and community physical activity environments consistently influenced all three types of FMS, including locomotor, ball, and composite skills. Additionally, seven individual-level factors (children’s age, sex, body mass index, light physical activity, sleep duration, perceived motor competence, and physical fitness) were associated with different types of FMS.

**Conclusions:**

The findings underscore the multidimensional and complex nature of FMS development, with individual-level factors playing a particularly significant role. Future research should adopt rigorous longitudinal designs, comprehensive assessment tools covering various FMS skills, and objective measurement of parents’ movement behaviors to better understand the strength and direction of the relationship between socio-ecological factors and children’s FMS.

**Supplementary Information:**

The online version contains supplementary material available at 10.1186/s12889-024-17728-2.

## Introduction

Fundamental motor skills (FMS) serve as the foundation for specialized movement sequences and sport skills that are essential for a wide range of activities, including playground games and organized sports [1, 2]. FMS can be categorized into three main groups: locomotor skills (e.g., running and jumping), object control skills or ball skills (e.g., throwing, catching and hitting), and stability skills (e.g., dynamic and static balance) [3, 4]. Proficiency in FMS plays a vital role in children’s physical, psychological and social development [5].

A substantial body of evidence has supported the association of greater proficiency in FMS with higher levels of physical activity (PA) and fitness and lower risks of overweight and obesity among children [4, 6]. Recent studies have also demonstrated the positive impact of FMS on children’s mental health outcomes, including perceived motor competence (PMC) [7, 8], well-being [9], and cognitive function [10, 11]. Despite the benefits of proficient FMS, research has indicated that the level of FMS in children around the world is low [12], and that Chinese children have significantly lower gross motor skills (e.g., aiming & catching tasks or throwing & catching tasks.) than their peers in countries such as the UK and US [13–15]. The underdevelopment in FMS not only adversely affects children’s physical, psychological, and social development during childhood [16, 17], but also diminishes motivation and engagement in physical activities during adolescence and adulthood, consequently increasing the risk of metabolic-related diseases (e.g., overweight and obesity) [18–20]. Therefore, it is crucial to implement effective strategies for promoting FMS in children. Identifying the factors associated with FMS that can be modified through interventions and policies is essential in this regard.

**Fig. 1:**
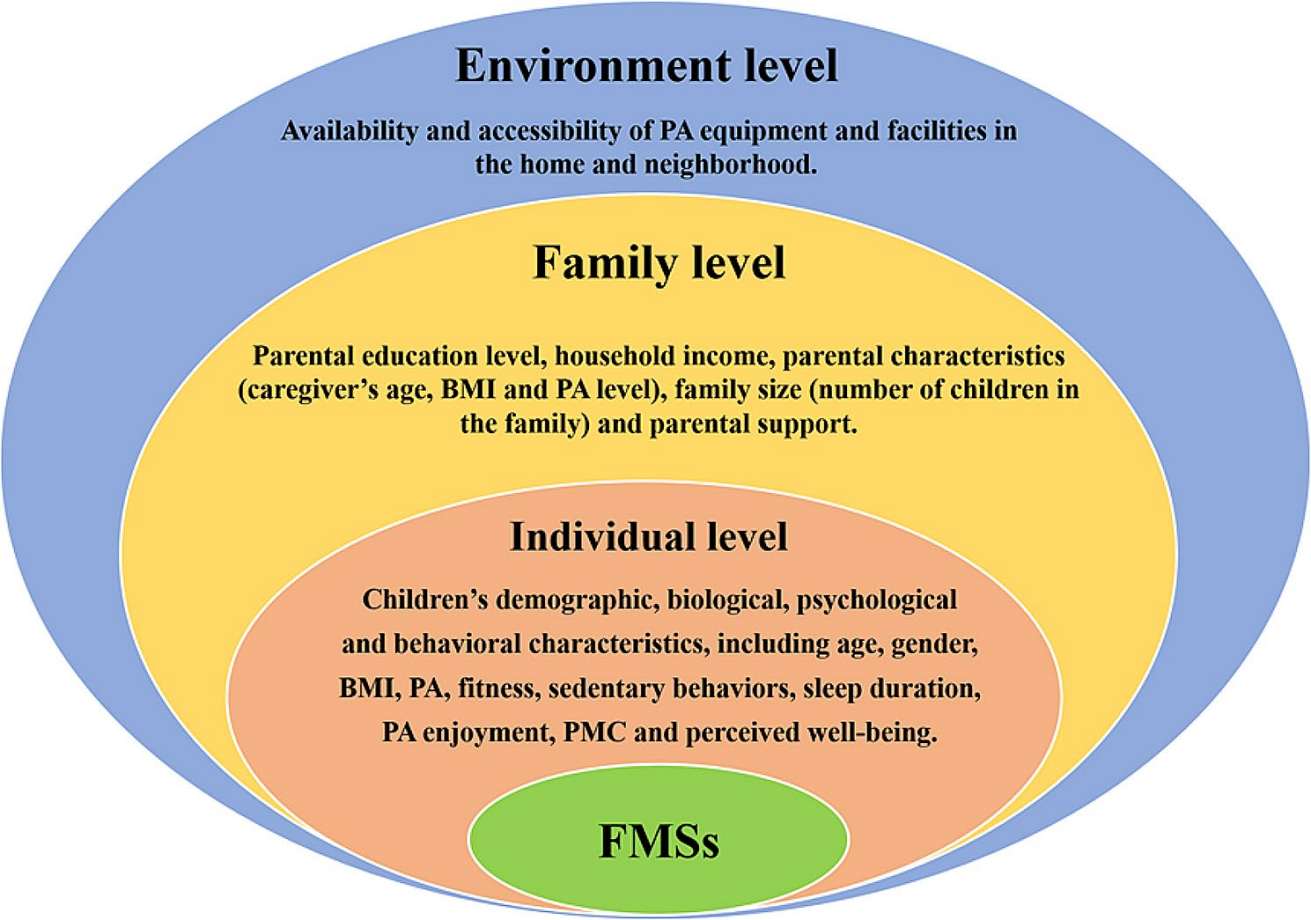
Social-ecological model for fundamental motor skills, PA = physical activity, PMC = perceived motor competence, BMI = body mass index, FMS = fundamental motor skills

Previous review studies have indicated that age, sex, and socioeconomic status are closely associated with children’s FMS. However, limited research has focused on exploring the interrelationship between psychological parental, social, and environmental factors and FMS among children [21]. To address this gap, social-ecological models of health behavior provide a theoretical framework through which potentially relevant factors can be conceptualized. These may include demographic and biological characteristics, psychological, cognitive and emotional traits, behavioral characteristics, social and cultural variables and environmental factors [22]. Given the association between PA and FMS, it is possible that the contextual factors that are associated with motor skill proficiency may be similar to those for PA [23]. According to the socio-ecological model, children’s FMS can be influenced by various factors and contextual characteristics at different levels, including: individual-level factors (e.g., children’s demographic, biological, psychological and behavioral characteristics), family-level factors (e.g., family socioeconomic status, parental characteristics, and parental support), and environmental-level factors (e.g., availability and accessibility of PA equipment and facilities in the home and neighborhood) (Fig. 1). By considering the socio-ecological model, researchers can explore the multifaceted influences on children’s FMS and gain a deeper understanding of their development.

Indeed, previous studies have conducted preliminary examinations of the association between social-ecological correlates and FMS in preschool and school-aged children. For instance, research has shown that various factors across individual, family, and environmental domains are linked with different aspects of FMS. Specifically, at the individual level, age, engagement in moderate-to-vigorous physical activity (MVPA), dance class attendance, physical fitness, and perceptual-motor coordination scores have been identified as being associated with FMS [23–25]. At the family level, parental physical activity, socioeconomic status (e.g., parental education level and income), parental support (e.g., frequency of purchasing equipment for their child), and number of children have been found to be positively related to motor skill performance [26–29]. At the environmental level, the presence of physical activity equipment and play spaces at home and in the neighborhood has been identified as a positive factor associated with the development of locomotor skills [24]. Although these studies have provided preliminary evidence for the relationship between certain individual and contextual characteristics and children’s FMS, there are still many potential correlates that have been understudied (e.g., caregiver’s characteristics). Additionally, most of the existing evidence regarding the correlates of FMS levels of children has been obtained from Western contexts, such as the US, Australia, and European countries, while evidence from Eastern countries is scarce [21]. To the best of our knowledge, there is a scarce of research examining the influential factors of children’s FMS from a social-ecological perspective in a Chinese context.

Therefore, the purpose of this study was to investigate the social-ecological correlates of FMS in Chinese school-aged children. Specifically, the study aimed to examine the association of individual-level factors (age, sex, body mass index [BMI], PA, sedentary behavior [SB], sleep duration [SLP], fitness, PA enjoyment, PMC, and perceived well-being), family-level factors (parental education level, household income, number of children in the family, parental support, caregiver’s age, BMI, and PA level), and environment-level factors (home and neighborhood PA equipment and facilities) with FMS (locomotor skills, ball skills, and composite skill) among Chinese school-aged children. The research findings will provide empirical evidence and serve as a foundation for promoting FMS in Chinese children and informing policy development in this area.

## Methods

### Research design and participants

This study employed a cross-sectional design, utilizing data obtained from the baseline survey of a large research project called the Fundamental motor skills Promotion Program for Obese Children, FMSPPOC). The entire project comprised a series of studies, encompassing population-based health surveillance among Chinese children (including both obese and non-obese individuals), health intervention for obese children, and exploration study of psychophysiological mechanisms. The FMSPPOC project was funded by the National Social Science Fund of China, specifically the National Office for Philosophy and Social Sciences in Beijing, China (Ref. No.: 19,200,526; 2019/20). Further details regarding the FMSPPOC project can be found elsewhere [87].

The minimal sample size for this study was determined using G*Power 3.1. With an a priori, two-tailed power calculation, an alpha level of 0.05, a statistical power of 80%, an effect size of *R*^2^ = 0.20 [23] and 22 predictive variables in the regression model, a total of 106 participants were required to ensure a robust statistical analysis. Accounting for an anticipated response rate of 80%, a minimum of 133 participants were recruited for this study. Eligible participants needed to meet specific inclusion criteria, which included: (1) no physical mobility restrictions (e.g., physical disabilities); (2) no cognitive and/or mental disorders; (3) no intellectual impairment and/or cardiovascular disease; (4) the children lived with their parents; and (5) the informed consent form was signed.

### Procedures and quality control

This study was conducted following the Declaration of the Helsinki World Medical Association [30]. The study protocol was approved by the Research Ethics Committee of authors’ affiliation (ref. No. 2021LLSC051), and it has been registered in Chinese Clinical Trial Registry (BLINDED; 25 Nov 2022). Using a random stratified sampling approach, six public primary schools (grade 1–6) were recruited in Shijiazhuang city, Hebei, China. For each school, two classes of students were randomly selected from each grade. Prior permission to conduct the study was obtained from the teachers and principals of the participating schools. All participants voluntarily took part in the study, and written informed consent was obtained from both the children and their parents before the study commenced.

Data collection was carried out by two experienced researchers with the assistance of the head teacher of each participating class, between 1 March and 15 April 2022. Objective measurements for height, weight, body composition, FMS, and physical fitness levels of the children were taken at the school’s sports center. These measurements were taken in the morning before class time, with groups being organized by class. Additionally, their levels of PA, SB, and SLP were objectively assessed. Demographic data (e.g., sex, date of birth, and ethnicity), as well as psychological variables (e.g., PMC, enjoyment, and perceived well-being), were collected through paper questionnaires (20–30 min/person). Furthermore, the primary caregiver of the children was invited to complete a package of paper questionnaires (15 min/person), which included information on parental demographics (e.g., sex, age, ethnicity, education level, number of children, and monthly household income), anthropometric data (e.g., height and weight), caregiver’s PA level, parental support for children’s PA behavior, and the home and neighborhood PA environment. Considering the limited cognitive ability of children in grades 1–2, the head teacher guided the students in completing the questionnaires (e.g., PMC, PA enjoyment, and perceived well-being) in the classroom. For children in grades 3–6, they were asked to independently complete the questionnaires before or after class, in a classroom setting. All the paper questionnaire responses were transferred to the Excel software for storage, which were subsequently analyzed in SPSS.

### Measurements

#### Individual-level correlates

Children’s demographic information, including age, sex, ethnicity, and medical conditions was collected through self-reported questionnaires. Following a standardized protocol [31], children’s body weight (kg) and body height (m^2^) were measured using calibrated medical digital scales (RGT-140, Changzhou, China) and a portable stadiometer (GMCS-I, Beijing, China) to the closest 0.05 kg and 0.1 cm, respectively. BMI was calculated as calculated as the ratio of body mass (kg) to the square of body height (m^2^). The Chinese sex-specific and age-specific BMI cutoffs points [32] were utilized to define overweight and obese participants.

The ActiGraph GT3X + accelerometer (ActiGraph LLC, Pensacola, FL, USA) was utilized to objectively measure the children’s daily PA. Each participant was instructed to wear the accelerometer on an elasticized belt at the right mid-axillary line, around the waist. The participants were encouraged to wear the accelerometer continuously for 24 h per day, removing it only during water-based activities such as swimming or bathing. The monitoring period lasted for a minimum of 7 days, including at least two weekend days. To ensure data validity, days with more than 16 h of activity recordings (from midnight to midnight) were considered valid [33]. Additionally, a minimum amount of non-sleep data was required for inclusion, which consisted of at least 4 days with at least 10 h of wake wear time per day, including at least one weekend day [34]. Data were collected at a sampling rate of 80 Hz downloaded in 1-second epochs using the ActiLife software version 6.13 (ActiGraph LLC). For analysis purposes, the data were reintegrated into 15-second epochs with the low-frequency extension filter applied [35]. Non-wear time was defined as a continuous period of 20 min or more with zero counts [36]. To determine time spent in different intensities of PA and sedentary time, Evenson cut-off points were utilized [36, 37]. Specifically, non-sleep time was classified as light PA (25–574 counts/15s), moderate PA (574–1003 counts/15s), vigorous PA (> 1003 counts/15s), and sedentary time as all movement ≤ 25 counts per 15 s. Night sleep duration was calculated using *R* software and the GGIR package (version 2.0) default algorithm, as described by previous studies [38]. Parents were instructed to fill in sleep logs for their child to cross-validate the waking (wear) time.

Physical fitness was assessed using the revised 2014 version of the Chinese National Student Physical Fitness Standard [39]. The definition, calculation and evaluation of comprehensive fitness in Chinese children and adolescents have been published previously in detail [40]. Briefly, seven components of physical fitness were included in our study, including BMI(a surrogate of body composition), Forced vital capacity(reflecting respiratory function and pulmonary function), 50 m sprint(reflecting explosive force and speed), Sit and reach(reflecting hamstring and lower back flexibility), Timed rope-skipping(a measure of motor coordination), Timed sit-ups (reflecting abdominal muscle strength, for grades 3–6 only), and 50 m×8 shuttle run (reflecting speed endurance for grades 5–6 only). Each fitness indicator score was weighted based on a grade- and sex-specific percentage, and a total physical fitness score was calculated. A higher score indicated a better level of physical fitness.

PMC of the children was assessed using the athletic competence subscale of the Self-perception Profile for Children (SPPC) [41]. The athletic competence subscale consists of six items, with three items reflecting low competence or adequacy and three items reflecting high perceptions of competence or adequacy. The scoring of the item is counterbalanced, with half of the items scored 1, 2, 3, 4, and the other half scored 4, 3, 2, 1. This counterbalancing ensures that children are attentively responding to the content of the items and not providing random or consistently biases responses [41]. The Chinese version of the SPPC has demonstrated adequate reliability, ranging from 0.67 to 0.76 [42]. Furthermore, the structure and criterion validity of the scale are acceptable (χ^2^/df = 2.69, CFI = 0.923, TLI = 0.917, RMSEA = 0.061) [43].

PA enjoyment was assessed using the revised Chinese edition of the Physical Activity Enjoyment Scale (PACES), which consists of seven items rated on a 5-point Likert scale ranging from 1 (“Disagree a lot”) to 5 (“Agree a lot”) [44]. All seven items derived from a modified 16-item version of PACES (Cronbach’ alpha = 0.87; (χ^2^/df = 2.12, CFI = 0.943, TLI = 0.936, RMSEA = 0.052) [45]. The scores for the seven items were reverse-coded and then averaged to calculate the overall score, with a higher score indicating greater enjoyment of physical activity.

Perceived well-being was measured using the Chinese version of the 12-item Psychological Well-Being Scale for Children (PWB-C) (Cronbach’ alpha = 0.91) [46]. PWB-C assesses six dimensions of psychological well-being: environmental mastery, personal growth, purpose in life, self-acceptance, autonomy, and positive relations with others. Participants provided responses on a 4-point Likert scale ranging from 1 (“almost never”) to 4 (“very frequently”) (χ^2^/df = 1.74, CFI = 0.952, TLI = 0.946, RMSEA = 0.052). The mean score of the 12 items was calculated, with a higher score indicating a higher level of perceived well-being.

#### Family-level correlates

Caregiver’s age, height and weight, number of children in the family, parental education level and monthly household income were self-reported [47]. Caregiver’s BMI was calculated as body mass (kg) divided by body height squared (m^2^). Additionally, caregiver’s PA was assessed using the Chinese version of the International Physical Activity Questionnaire-short form (IPAQ-S), which has been validated in Chinese adults (ICC = 0.74) [48].

Parents’ support for children’s PA behavior was measured using the six items adapted from a study by Rhodes et al. [49]. Three items were used to assess parental support for children’s MVPA (e.g., “How often per week do you encourage your child to participate in MVPA”), while another three items were used to measure parental support for children’s light-intensity PA (LPA) (e.g., “How often per week do you engage in light physical activities together with your child”). Participants rated their responses on a 5-point Likert scale, ranging from 1(“never/rarely”) to 5(“almost every day”) (Cronbach’s α = 0.75 for MVPA and α = 0.80 for LPA) [49]. The mean score of six items were calculated, with a higher score indicating a greater level of parental support for children’s PA.

#### Environment-level correlates

The environment-level correlates were measured using adapted items from the Neighborhood Impact on Kids (NIK) study survey (Cronbach’ alpha = 0.84) [50]. The questionnaire included eight types of play equipment and sports facilities commonly found in homes and communities (e.g., bicycles, basketball racks, jump ropes, active video games, various balls/rackets, swimming pools, roller skates/skateboards and swings/playhouses/jungle gyms). Parents were asked to indicate how frequently their children used these devices/facilities on a 5-point Likert scale, ranging from 1(“no or not available”) to 5 (“at least once a week or more”) (χ^2^/df = 2.11, CFI = 0.931, TLI = 0.929, RMSEA = 0.053). The average score of all items were calculated, with a higher score indicating a more favorable PA environment in children’s home and neighborhood.

#### FMS

FMS was assessed using the Test of Gross Motor Development-Third Edition (TGMD-3) which has been validated in China and has demonstrated satisfactory inter-rater (ICC = 0.87) and intra-rater reliability (ICC = 0.95) [51]. The TGMD-3 consists of two sub-scales: the locomotor skill sub-scale, which includes six skills (run, gallop, hop, horizontal jump, slide, and skip) (inter-rater = 0.83, intra-rater = 0.93), and the ball skill sub-scale, which includes seven skills (one hand forehand strike of a self-bounced tennis ball, kick a stationary ball, overhand throw, underhand throw, two hand strike of a stationary ball, one hand stationary dribble, and two hand catch) (inter-rater = 0.79, intra-rater = 0.93). Each child performed three trials, with one practice trial followed by two formal trials. Only the scores from the two formal trials were recorded for evaluation. Children’s performances were observed and evaluated based on 3–5 qualitative performance criteria for each TGMD-3 assessment skill, with each criterion scored as either 1 point (present) or 0 points (absent) using process-oriented checklists [52]. The entire testing process was recorded simultaneously by two cameras (SONYHDR-CX680, China), and the recorded videos were independently scored by two experienced evaluators. In cases where there was a significant difference in scores between the two evaluators, a senior scientific research supervisor intervened to reach a consensus. The raw score for each item was the sum of the scores from both trials. The sums of the items were used to calculate the raw scores for the locomotor (maximum score of 46) and ball skills sub-scales (maximum score of 54), as well as the overall TGMD-3 score (maximum score of 100) [52]. The TGMD-3 indicated a good construct validity in this study, with χ^2^/df = 103.28, CFI = 0.960, TLI = 0.952, RMSEA = 0.043.

### Statistical analyses

Data analysis was performed using SPSS 27.0 (IBM Corp., Armonk, NY, USA). Prior to the main analysis, missing values and outliers (defined as values exceeding ± 3 standard deviations from the mean) for all variables were addressed. Descriptive statistics were calculated, presenting continuous variables as mean ± standard deviation (M ± SD) and discrete variables as frequency (%). To examine the association between social-ecological factors and FMS, multiple-level regression models were employed. In Model 1, individual-level factors such as children’s sex, age, BMI, fitness, LPA, MVPA, SB, SLP, PMC, PA enjoyment, and perceived well-being were included as predictors. Model 2 incorporated family-level factors, including parental education, household income, number of children, parental support for children’s PA behavior, caregiver’s age, BMI, and PA behaviors. Model 3 expanded the analysis to include neighborhood and home PA environmental variables as predictors. To assess the magnitude of the associations between predictors, effect size of Cohen *f*^*2*^ was calculated, with values of 0.02, 0.15, and 0.35 indicating small, medium, and large effects, respectively [53]. The significance level for all analyses was set at *P* < 0.05 (two-tailed).

## Results

### Sample characteristics

**Fig. 2:**
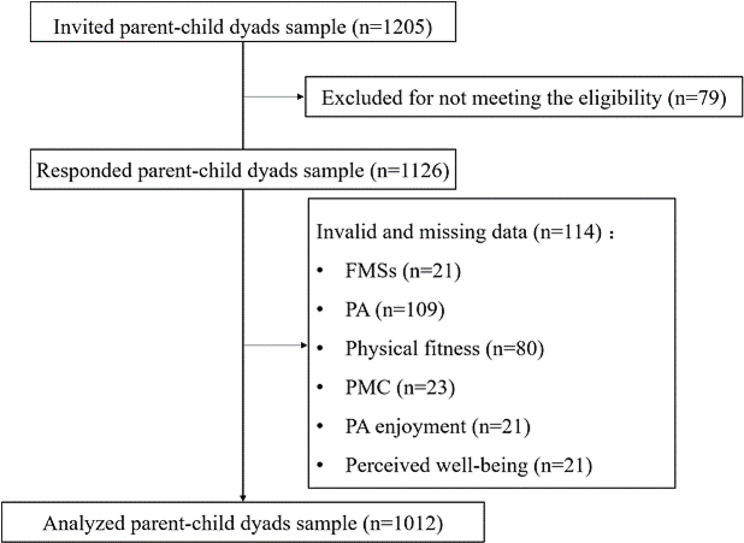
STROBE diagram of study process; FMS = fundamental motor skills, PA = physical activity, PMC = perceived motor competence

A total of 1205 parent-child dyads were contacted to participate in the study, and the questionnaire response rate was 93.3%. After eliminating invalid and outlier values, data of 1012 parent-child dyads were finally included for statistical analysis (Fig. 2). At the individual level, among the 1102 children (9.39 ± 1.15 years, 47.1% girls), 13.5% and 17.6% were classified as overweight and obese, respectively. At the family level, 47.1% of the children’s caregivers were mothers (37.36 ± 4.75 years); 30.3% of fathers and 38.9% of mothers had college degrees or above; the proportion of households with middle or higher income was 51.0%. The number of children in the families varied, with most families having 2–4 children (60.7%). More details of the participants characteristics can be found in Appendix 1.

### Correlates of FMS

For locomotor skills, the multi-level regression model showed that five out of 11 individual-level factors significantly predicted locomotor skills (*R*^2^ = 0.125, *P* < 0.001; see Table 1, Model 1), including age (*β* = 0.19, *P*<0.001), BMI (*β*= -0.17, *P* < 0.001), LPA (*β* = 0.11, *P* < 0.01), PMC (*β* = 0.06, *P* < 0.05), fitness (*β* = 0.15, *P* < 0.001). Among family factors, only parental support for children’s PA participation (*β* = 0.30, *P <* 0.001) positively predicted locomotor skills after controlling for individual factors (*R*^2^ = 0.212, *P* < 0.001; see Table 1, Model 2). In addition, after controlling for family and individual factors, neighborhood and home PA environment (*β* = 0.32, *P* < 0.001) significantly positively predicted locomotor skills (*R*^2^ = 0.293, *P* < 0.001; see Table 1, Model 3).

**Table 1:** Hierarchical regression predicting children’s locomotor skills from social-ecological correlates

Variable	Model 1		Model 2		Model 3	
	B (95%CI)	β	B (95%CI)	β	B (95%CI)	β
Individual-level correlates						
Age (years)	0.56 [0.37, 0.76]	0.19 ***	0.43 [0.23, 0.62]	0.14 ***	0.69 [0.50 0.88]	0.23 ***
Sex						
Girls	-0.34 [-0.91, 0.23]	-0.04	-0.45 [-1.00, 0.09]	-0.05	-0.92 [-1.44, -0.39]	-0.10 ***
Boys	Reference		Reference		Reference	
BMI (kg/m^2^)	-0.21 [-0.29, -0.13]	-0.17 ***	-0.20 [-0.28, -0.13]	-0.17 ***	-0.20 [-0.27, -0.13]	-0.16 ***
LPA (mins/day)	0.01 [0.00, 0.01]	0.11 **	0.01 [0.00, 0.01]	0.10 **	0.01 [0.00, 0.01]	0.07 *
MVPA (mins/day)	0.01 [-0.02, 0.03]	0.02	-0.01 [-0.03, 0.02]	-0.02	-0.00 [-0.02, 0.02]	-0.01
Sedentary behavior time (hours/day)	0.10 [-0.01, 0.30]	0.04	0.06 [-0.14, 0.25]	0.02	0.04 [-0.14, 0.22]	0.02
Sleep duration (hours/day)	-0.34 [-0.70, 0.02]	-0.06	-0.22 [-0.56, 0.13]	-0.04	-0.17 [-0.49, 0.16]	-0.03
Perceived motor competence	0.53 [0.00, 1.05]	0.06 *	0.23 [-0.28, 0.74]	0.03	0.19 [-0.29, 0.67]	0.02
PA enjoyment	0.34 [-0.01, 0.70]	0.06	0.29 [-0.05, 0.64]	0.05	0.21 [-0.12, 0.53]	0.04
Wellbeing	-0.11 [-0.77, 0.55]	-0.01	-0.16 [-0.79, 0.47]	-0.01	-0.19 [-0.79, 0.41]	-0.02
Physical fitness	0.06 [0.03, 0.09]	0.15 ***	0.04 [0.02, 0.07]	0.11	0.04 [0.02, 0.07]	0.11 ***
Family-level correlates						
Father education level						
Below college			-0.23 [-0.82, 0.36]	-0.02	-0.27 [-0.82, 0.29]	-0.03
College and above			Reference		Reference	
Mother education level						
Below college			-0.47 [-1.04, 0.09]	-0.05	-0.53 [-1.07, 0.00]	-0.06
College and above			Reference		Reference	
Monthly household income						
Low income			-0.03 [-0.88, 0.83]	-0.00	-0.13 [-0.94, 0.68]	-0.01
Medium income			-0.15 [-1.01, 0.71]	-0.02	-0.25 [-1.06, 0.57]	-0.03
High income			Reference		Reference	
Caregiver Age (years)			-0.00 [-0.06, 0.06]	-0.00	-0.01 [-0.06, 0.04]	-0.01
Caregiver BMI (kg/m^2^)			-0.04 [-0.09, 0.01]	-0.04	-0.04 [-0.08, 0.01]	-0.04
Caregiver LPA (mins/day)			-0.00 [-0.02, 0.01]	-0.01	-0.01 [-0.02, 0.01]	-0.02
Caregiver MVPA (mins/day)			0.00 [-0.01, 0.02]	0.01	0.00 [-0.01, 0.02]	0.01
Child numbers			0.11 [-0.32, 0.55]	0.02	0.00 [-0.41, 0.41]	0.00
Parental support for children’s PA			1.67 [1.34, 1.99]	0.30 ***	1.31 [0.99, 1.62]	0.24 ***
Environment correlates						
Home equipment/space					2.61	0.32 ***
*R* ^*2*^	0.125		0.212		0.293	
*ΔR* ^*2*^			**0.087**		**0.081**	
Effect size (*f*^*2*^ )	0.143		0.269		0.414	

*Note*: *P < 0.05; **P < 0.01; ***P < 0.001

For ball skills, the multi-level regression model showed that five out of 11 individual-level factors significantly predicted ball skills (*R*^2^ = 0.278, *P* < 0.001; see Table 2, Model 1), including age (*β* = 0.22, *P* < 0.01), sex (*β* = 0.41, *P* < 0.001), sleep duration (*β*= -0.12, *P* < 0.001), PMC (*β* = 0.12, *P* < 0.001) and fitness (*β* = 0.18, *P* < 0.001). Among family factors, only parental support for children’s PA participation (*β* = 0.28, *P* < 0.001) positively predicted ball skills after controlling for individual factors (*R*^2^ = 0.354, *P* < 0.001; see Table 2, Model 2). In addition, after controlling for family and individual factors, neighborhood and home PA environment (*β* = 0.27, *P* < 0.001) significantly positively predicted ball skills (*R*^2^ = 0.413, *F*(22, 988) = 31.581, *P* < 0.001, see Table 2, Model 3).

**Table 2:** Multi-level linear regression predicting children’s ball skills from social-ecological correlates

	Model 1		Model 2		Model 3	
	B (95%CI)	*β*	B (95%CI)	*β*	B (95%CI)	*β*
Individual correlates						
Age (years)	0.86 [0.62, 1.10]	0.22 ***	0.75 [0.52, 0.99]	0.19 ***	1.05 [0.82, 1.28]	0.26 ***
Sex						
Girls	4.89 [4.20, 5.58]	0.41 ***	4.78 [4.12, 5.44]	0.40 ***	4.25 [3.61, 4.89]	0.35 ***
Boys	Reference		Reference		Reference	
BMI (kg/m^2^)	0.06 [-0.04, 0.16]	0.04	0.05 [-0.14, 0.15]	0.03	0.06 [-0.03, 0.15]	0.03
LPA (mins/day)	0.00 [-0.00, 0.01]	0.02	0.00 [-0.01, 0.01]	0.01	-0.00 [-0.01, 0.00]	-0.01
MVPA (mins/day)	-0.01 [-0.03, 0.02]	-0.02	-0.02 [-0.05, 0.01]	-0.05	-0.02 [-0.04, 0.01]	-0.04
Sedentary behavior time (hours/day)	-0.10 [-0.34, 0.14]	-0.03	-0.14 [-0.37, 0.10]	-0.04	-0.15 [-0.37, 0.07]	-0.04
Sleep duration (hours/day)	-0.87 [-1.31, -0.44]	-0.12 ***	-0.68 [-1.10, -0.26]	-0.10 **	-0.62 [-1.22, -0.22]	-0.09 **
Perceived motor competence	1.33 [0.69, 1.97]	0.12 ***	0.93 [-0.31, 1.54]	0.08 **	0.88 [0.30, 1.47]	0.08 **
PA enjoyment	0.43 [-0.00, 0.87]	0.06	0.39 [-0.03, 0.80]	0.05	0.29 [-0.11, 0.68]	0.04
Wellbeing	-0.03 [-0.83, 0.77]	-0.00	-0.16 [-0.92, 0.61]	-0.01	-0.19 [-0.92, 0.54]	-0.01
Physical fitness	0.10 [0.06, 0.13]	0.18 ***	0.08 [0.05, 0.11]	0.14 ***	0.08 [0.05, 0.10]	0.14 ***
Family correlates						
Father education level						
Below college			-0.11 [-0.82, 0.61]	-0.01	-0.15 [-0.83, 0.53]	-0.01
College and above			Reference		Reference	
Mother education level						
Below college			-0.13 [-0.81, 0.56]	-0.01	-0.20 [-0.85, 0.46]	-0.02
College and above			Reference		Reference	
Monthly household income						
Low income			-0.90 [-1.94, 0.13]	-0.08	-1.02 [-2.01, -0.03]	-0.09 *
Medium income			-0.85 [-1.89, 0.19]	-0.07	-0.96 [-1.95, 0.03]	-0.08
High income			Reference		Reference	
Caregiver Age (years)			-0.03 [-0.10, 0.14]	-0.03	-0.04 [-0.11, 0.02]	-0.03
Caregiver BMI (kg/m^2^)			0.02 [-0.04, 0.08]	0.01	0.02 [-0.04, 0.08]	0.02
Caregiver LPA (mins/day)			0.00 [-0.02, 0.02]	0.00	-0.00 [-0.02, 0.01]	-0.01
Caregiver MVPA (mins/day)			0.01 [-0.01, 0.02]	0.02	0.01 [-0.01, 0.02]	0.02
Child numbers			0.36 [-0.16, 0.89]	0.04	0.23 [-0.27, 0.74]	0.02
Parental support for children’s PA			2.07 [1.68, 2.46]	0.28 ***	1.66 [1.28, 2.04]	0.23 ***
Environment correlates						
Home equipment/space					2.97 [2.38, 3.55]	0.27 ***
*R* ^*2*^	0.278		0.354		0.413	
*ΔR* ^*2*^			0.076		0.059	
Effect size (*f*^*2*^ )	0.385		0.548		0.704	

*Note*: *P < 0.05; **P < 0.01; ***P < 0.001

For composite skills (TGMD-3 score), the multi-level regression model showed that seven out of 11 individual-level factors significantly predicted composite skills (*R*^2^ = 0.234, *P* < 0.001; see Table 3, Model 1), including age (*β* = 0.25, *P* < 0.001), sex (*β* = 0.27, *P* < 0.001), BMI (*β*= -0.07, *P* < 0.05), LPA (*β* = 0.07, *P* < 0.05), sleep duration (*β*= -0.13, *P* < 0.001), PMC (*β* = 0.12, *P* < 0.001) and fitness (*β* = 0.21, *P* < 0.001). Among family factors, only parental support for children’s PA participation (*β* = 0.36, *P* < 0.001) positively predicted composite skills after controlling for individual factors (*R*^2^ = 0.355, *P* < 0.001; see Table 3, Model 2). Additionally, after controlling for family and individual factors, neighborhood and home PA environment (*β* = 0.36, *P* < 0.001) significantly positively predicted ball skills (*R*^2^ = 0.462, *P* < 0.001).

**Table 3:** Multi-level linear regression predicting children’s composite skills (TGMD-3 score) from social-ecological correlates

	Model 1		Model 2		Model 3	
	B (95%CI)	*β*	B (95%CI)	*β*	B (95%CI)	*β*
Individual correlates						
Age (years)	1.42 [1.08, 1.76]	0.25 ***	1.18 [0.85, 1.51]	0.21 ***	1.74 [1.43, 2.05]	0.31 ***
Sex						
Girls	4.56 [3.57, 5.55]	0.27 ***	4.35 [3.42, 5.27]	0.26 ***	3.35 [2.50, 4.21]	0.20 ***
Boys	Reference		Reference		Reference	
BMI (kg/m^2^)	-0.15 [-0.30, -0.01]	-0.07 *	-0.16 [-0.29, -0.03]	-0.07 *	-0.15 [-0.27, -0.03]	-0.07 *
LPA (mins/day)	0.01 [0.00, 0.02]	0.07 *	0.01 [-0.00, 0.02]	0.06	0.00 [-0.00, 0.01]	0.03
MVPA (mins/day)	-0.00 [-0.04, 0.04]	-0.00	-0.03 [-0.07, 0.01]	-0.05	-0.02 [-0.06, 0.02]	-0.04
Sedentary behavior time (hours/day)	-0.01 [-0.36, 0.34]	-0.00	-0.09 [-0.42, 0.23]	-0.02	-0.12 [-0.42, 0.17]	-0.02
Sleep duration (hours/day)	-1.27 [-1.90, -0.64]	-0.13 ***	-0.95 [-1.54, -0.36]	-0.10 **	-0.84 [-1.38, -0.30]	-0.09 **
Perceived motor competence	1.86 [0.94, 2.78]	0.12 ***	1.17 [0.31, 2.03]	0.08 **	1.09 [0.30, 1.87]	0.07 **
PA enjoyment	0.76 [0.14, 1.39]	0.07	0.67 [0.09, 1.25]	0.06	0.48 [-0.05, 1.01]	0.05
Wellbeing	-0.21 [-1.37, 0.94]	-0.01	-0.39 [-1.46, 0.68]	-0.02	-0.45 [-1.43, 0.52]	-0.02
Physical fitness	0.16 [0.11, 0.20]	0.21 ***	0.12 [0.08, 0.16]	0.16 ***	0.12 [0.08, 0.16]	0.16 ***
Family correlates						
Father education level						
Below college			-0.24 [-1.23, 0.76]	-0.01	-0.32 [-1.23, 0.59]	-0.02
College and above			Reference		Reference	
Mother education level						
Below college			-0.62 [-1.58, 0.33]	-0.04	-0.76 [-1.63, 0.12]	-0.04
College and above			Reference		Reference	
Monthly household income						
Low income			-0.90 [-2.35, 0.54]	-0.05	-1.13 [-2.45, 0.20]	-0.07
Medium income			-1.05 [-2.51, 0.40]	-0.06	-1.26 [-2.59, 0.07]	-0.07
High income			Reference		Reference	
Caregiver Age (years)			-0.04 [-0.13, 0.06]	-0.02	-0.06 [-0.14, 0.03]	-0.03
Caregiver BMI (kg/m^2^)			-0.01 [-0.01, 0.07]	-0.01	-0.01 [-0.08, 0.07]	-0.00
Caregiver LPA (mins/day)			-0.00 [-0.03, 0.02]	-0.00	-0.01 [-0.03, 0.01]	-0.02
Caregiver MVPA (mins/day)			0.01 [-0.02, 0.03]	0.02	0.01 [-0.02, 0.03]	0.02
Child numbers			0.49 [-0.24, 1.22]	0.04	0.25 [-0.42, 0.92]	0.02
Parental support for children’s PA			3.70 [3.16, 4.25]	0.36 ***	2.93 [2.42, 3.44]	0.29 ***
Environment correlates						
Home equipment/space					5.58 [4.80, 6.37]	0.36 ***
*R* ^*2*^	0.234		0.355		0.462	
*ΔR* ^*2*^			0.121		0.107	
Effect size (*f*^*2*^ )	0.305		0.550		0.859	

*Note*: *P < 0.05; **P < 0.01; ***P < 0.001

## Discussion

Throughout childhood, the development of FMS plays a crucial role in fostering an active and healthy lifestyle and contributes significantly to children’s overall development ^[4]^. Drawing upon the socio-ecological framework, this study aimed to explore the associations between individual, family, and environmental factors and FMS among Chinese school-aged children. The findings of our study revealed several consistent correlates of FMS across all three types (locomotor, ball, and composite skills) among children. These included age, sex, physical fitness, parental support, and the quality of home and community PA environments. Furthermore, we identified seven individual-level factors that were associated with different types of FMS in children.

### Individual-level correlates of FMS

#### Age

In our study, age emerged as a significant correlate of different types of FMS, aligning with the findings of Barnett et al., which indicated that motor skills develop with age [21]. From a developmental psychology perspective, the acquisition of FMS is not solely driven by natural development and maturation but also by continuous interaction with a stimulating and supportive social and physical environment [17]. Previous research has reported that older children often have more exposure to a variety of specialized sports, such as track and field, football, basketball, and baseball, compared to younger children. This increased exposure allows for steady improvement through repeated practice and reinforcement in sports [54]. Furthermore, as children grow older, their brain development gradually matures, leading to the development of motor learning ability and cognitive function [55]. It is worth noting that the participants involved in this study are currently in the phase of developing fundamental movement skills (mean age = 9.39 ± 1.15 years) and have not yet reached the stage of mastery expected at the age of 10, according to the Triangulated Hourglass Model [56]. Additionally, considering the expected ceiling effect during early to mid-adolescence [57], it is not surprising to find a notable correlation between age and motor skills among the participants.

#### Sex

Regarding sex, our findings revealed a positive correlation between girls and locomotor skills, while boys showed a positive correlation with ball skills. These findings are consistent with previous evidence [21, 58, 59]. The sex differences in FMS can be attributed to sport preferences and social environmental factors [60]. Previous studies have shown that the types of activities boys and girls engage in are strongly influenced by family, peers, teachers, and the physical environment [61]. Generally, boys are more inclined to participate in ball games, while girls tend to prefer dance and gymnastics [62]. Additionally, boys may receive more support, encouragement, and opportunities to engage in PA in school, family, and community settings compared to girls [21]. Importantly, when locomotor and ball skills were combined to assess composite skills, boys showed a positive correlation with composite skills. These results underscore the importance of focusing on developing girls’ ball skills. Therefore, future intervention studies aimed at promoting the development of FMS in children should pay special attention to sex differences in different types of motor skills to develop more effective and targeted promotion programs.

#### Weight status

In our study, we found a significant negative correlation between BMI and children’s locomotor and composite skills, but no significant correlation with ball skills, which is consistent with previous findings [21]. The negative correlation between BMI and locomotor skills can be attributed to the mechanical constraints experienced by children with higher body mass when performing locomotor and stability tasks [63]. For overweight and obese children, the excess body mass imposes additional strain and burden on the skeletal and muscular systems, hindering functional movement, particularly in tasks that involve moving or advancing body mass [64]. Interestingly, no significant correlation between BMI and ball skills was observed in our study. This may be explained by the fact that all participants, regardless of their weight status, demonstrated poor levels of ball skills, making it challenging to discern detectable differences (i.e., a floor effect). This explanation is supported by a recent systematic review of FMS data from 21,000 children aged 3 to 10 years in 25 countries on 6 continents, which revealed that very few children performed well on tests of object control/ball skills [12]. In addition, cultural differences may provide another explanation for the lack of association between BMI and ball skills. The TGMD-3 (as well as its previous versions, TGMD and TGMD-2) is an evaluation tool for FMS developed within the cultural context of the United States [12]. Ball skills assessed by the TGMD-3 are associated with popular sports in the US, such as baseball, basketball, and American football. However, in China, children may have greater exposure to small-ball education, such as table tennis and badminton. As a result, ball skills evaluated within the TGMD-3 assessment system may not receive adequate and targeted guidance and practice for Chinese children, leading to a low overall level of proficiency. In the future, it would be worthwhile to develop assessment tools suitable for local children, taking into account the Chinese cultural background and sports characteristics.

#### PA level

In the final model, we observed that only LPA was associated with locomotor skills, while neither LPA nor MVPA showed associations with ball skills and composite skills among children. This finding contradicts previous studies that have examined the relationship between PA intensity (the outcome or dependent variable) and FMS. Previous results have consistently shown that moderate-to-vigorous physical activity (MPA) and vigorous physical activity (VPA) are usually associated with FMS, while light physical activity (LPA) is not [65, 66]. However, our study included children from the entire school-age range (7 to 12 years), which may explain the inconsistency. As postulated in Stodden et al.’ s model, early childhood experiences with PA play a crucial role in the development of FMS, and proficiency in FMS becomes more important for sustained PA participation over time [67]. It is important to note that the relationship between PA and FMS may not be direct and may be influenced by PMC and levels of physical fitness [21, 68]. In a recent review, Barnett et al. also highlighted the insufficient evidence supporting the pathway from PA to FMS [59]. It is necessary to examine the association between specific types of PA and specific dimensions of FMS within the context of children’s daily life scenarios. In the future, rigorous longitudinal designs are needed to investigate the correlation between FMS and PA (intensity, type, and total volume) in children, providing empirical evidence for promoting the healthy development of children.

#### Fitness level

Regarding fitness levels, our study revealed that higher levels of physical fitness were associated with better FMS, including locomotor, ball, and composite skills, which is consistent with previous findings [2]. Previous research has suggested that although FMS and physical fitness are theoretically distinct constructs, they are closely intertwined. On one hand, numerous motor and fitness tasks require a high degree of neuromuscular control (e.g., motor unit recruitment, optimal co-activation of agonist/antagonist muscles) for efficient and coordinated movement [69]. On the other hand, there are overlapping tests in both FMS and physical fitness assessment programs, such as the standing long jump and running [2]. Furthermore, learning and mastering any motor skill require repetitive practice, which not only enhances musculoskeletal fitness but can also positively influence cardiorespiratory fitness [70]. This may partly explain the positive correlation between physical fitness and FMS. It is important to note that Barnett et al., in their latest review, emphasized that although there is strong evidence supporting the positive prediction of physical fitness (FMS→physical fitness) by FMS, the evidence supporting the reverse path (physical fitness→FMS) is very limited [59]. Physical fitness is a comprehensive concept, and the assessment tools used in the literature (e.g., single tests or comprehensive tests) are highly heterogeneous, making it challenging to analyze and compare research findings. Therefore, rigorous longitudinal designs should be employed in future studies to further explore the strength and direction of the relationship between FMS and physical fitness.

#### PMC

We observed a positive association of PMC with ball skills and composite skills, yet not with locomotor skills, which aligns with the findings of Rogers et al. [71] in female adolescents. However, previous studies have reported mixed results regarding the relationship between PMC and FMS among children [72–74]. Recent reviews have emphasized that the relationship between PMC and FMS remains unclear and may be influenced by various factors, such as sociocultural factors, cognitive function, motivation for PA participation, and consistency between FMS and PMC measurements [59, 75]. To address this issue, more rigorous longitudinal studies are needed in the future.

#### SLP and SB

Regarding SLP and SB, our final model indicated a positive association of SLP with ball skills and composite skills, which is consistent with previous research [76, 77]. One possible explanation for these findings is that sleep plays a crucial role in neuroplasticity processes, facilitating memory consolidation and contributing to motor skill learning and development [78]. However, our study did not find any association between SB and FMS, which aligns with the findings of Cliff et al. [79] and Graf et al. [80]. A recent review by Santos et al. suggested a negative correlation between SB and FMS, proposing that elementary school students’ movement behaviors are relatively stable and interact with each other. An increase in one behavior time (e.g., SB) leads to a decrease in another behavior time (e.g., PA), thus reducing the child’s opportunities for developing FMS [81]. Given the mixed results mentioned previously, future research need to further examine the longitudinal associations and underlying mechanisms between 24-hour movement behavior and FMS within the framework of time-use epidemiology [82].

### Family- and environment-level correlates of FMS

At the family level, we found a positive correlation between parental support and FMS, indicating that greater parental support is associated with better motor development in children. This finding aligns with previous research confirming that both direct and indirect parental support, such as engaging in physical activities with children, providing transportation support, purchasing toys and sports equipment, and encouraging children to exercise, are positively correlated with children’s PA behavior [83]. These forms of support provide children with more opportunities to engage in structured and unstructured activities, allowing them to practice FMS and improve their proficiency over time. Interestingly, our study did not reveal a significant correlation between parental education level, household income, and children’s FMS, which contradicts prior research [24, 27]. This discrepancy could be explained by variances in population demographics and social context. Previous investigations were conducted in Western countries where higher levels of parental education and family income are commonly linked to better FMS in children. This is largely due to parents being more likely to provide sufficient emotional and financial support (e.g., positive atmosphere, more access to sports equipment and opportunities), which in turn, bolsters PA and FMS of children [21]. However, this study focused on a sample of individuals living in the Chinese region, where economic inequalities among participants are less pronounced. The dissimilarities in population demographics and social context mentioned earlier, may also explain the inconsistent results between our study and Rodrigues et al.‘s study [28] regarding the association between family size (i.e., number of children in family) and children’s FMS. Although Rodrigues et al.‘s study [28] suggested that children in families with siblings, regardless of age and gender, exhibited better FMS development, our study’s unique population and social context could have played a role in the divergence of outcomes. Further research is needed to better understand the relationship between socioeconomic status, family structure, and FMS in diverse populations and social context.

Additionally, although we did not find an association between caregiver’s PA levels/BMI and children’s FMS in our study, previous research has demonstrated that fathers’ PA levels are positively correlated with their children’s FMS [84], and children of overweight/obese parents may be at risk for motor delays [85]. Therefore, future studies on promoting FMS should also consider the potential effects of parents’ PA behavior and characteristics on children’s motor development.

At the environmental level, we found a positive association between the frequency of using home and neighborhood play equipment/sports facilities and children’s FMS, consistent with previous findings [23, 24]. Existing evidence suggests that a supportive PA environment in the proximity of the home and neighborhood is associated with increased MVPA and decreased SB in children [86]. When children have access to adequate play equipment/sports facilities and spacious areas in their home and neighborhood, they are more likely to engage in activities that provide repeated opportunities to practice and enhance FMS, thereby improving their proficiency in these skills. This highlights the importance of fostering a supportive PA environment around the home and neighborhood to promote the overall healthy development of children.

### Strengths and limitations of the study

The study has several strengths that should be highlighted. Firstly, it assessed a wide range of individual, family, and environmental correlates of FMS based on the socio-ecological model, providing a comprehensive understanding of potential risk factors that can be modified through intervention programs. Secondly, the study identified empirical evidence for the formulation of future intervention programs by examining these correlates. However, there are also several limitations should be noted. Firstly, it should be noted that there are certain limitations with the sample that may affect its representativeness. Specifically, the exclusion of individuals with physical or intellectual disabilities, combined with a low level of parental education, and the fact that it was derived solely from a single large city in northern China, could curtail the generalizability of the findings to other populations and contexts. Secondly, the FMS assessment tool (TGMD-3) used in this study only included locomotor and ball skills, neglecting stability skills such as balance. Therefore, the relationship between the examined factors and stability skills remains unknown. Thirdly, data collection occurred during the novel coronavirus epidemic, and the implemented epidemic prevention and control policies may have influenced children’s outdoor activities, potentially masking the true relationship between PA and FMS. Lastly, the cross-sectional nature of the study restricts the ability to establish causality between the examined factors and FMS.

## Conclusions

In conclusion, this study provides valuable insights into the socio-ecological correlates of FMS among school-aged children in China. The findings highlight the multidimensional and complex nature of factors influencing FMS development, which vary slightly depending on the type of skill. Individual-level factors appear to be particularly influential. Future research should employ rigorous longitudinal designs, utilize comprehensive FMS assessment tools covering locomotor, ball, and stability skills, and objectively measure parents’ PA behaviors to further elucidate the strength and direction of the relationship between socio-ecological factors and children’s FMS.

### Electronic supplementary material

Below is the link to the electronic supplementary material.

Supplementary Material 1

## Data Availability

The dataset supporting the conclusions of this article will be available from the corresponding author upon reasonable request.
