# Supplementary material for: Individual, family, and environmental correlates of fundamental motor skills among school-aged children: a cross-sectional study in China
Source: BMC Public Health. 2024 Jan 17;24:208. doi: 10.1186/s12889-024-17728-2 (PMC10795326; doi:10.1186/s12889-024-17728-2)
Supplement: Supplementary file 1 — Supplementary Material 1 [file 12889_2024_17728_MOESM1_ESM.docx]

**Appendix 1 Descriptive characteristics of the study sample**

| **Variable** | All (n=1012) | Boys (n=535) | Girls (n=477) |
| --- | --- | --- | --- |
| **Individual-level correlates** |  |  |  |
| Age (years) | 9.39±1.51 | 9.41±1.51 | 9.36±1.51 |
| BMI (kg/m^2^) | 17.27±3.68 | 17.78±3.86 | 16.69±3.36 |
| Non-overweight/obesity | 679 (68.9%) | 334(62.4%) | 363(76.1%) |
| Overweight | 137 (13.5%) | 92(17.2%) | 45(9.4%) |
| Obesity | 178 (17.6%) | 109(20.4%) | 69(14.5%) |
| Child LPA (mins/day) | 184.04±58.07 | 191.99±59.48 | 175.13±55.18 |
| Child MVPA (mins/day) | 31.71±15.34 | 35.96±16.84 | 26.93±11.77 |
| SB (hours/day) | 4.65±1.66 | 4.48±1.68 | 4.84±1.61 |
| SLP (hours/day) | 9.01±0.85 | 8.99±0.87 | 9.02±0.82 |
| Physical fitness score | 72.48±11.28 | 70.48±11.91 | 74.71±10.08 |
| Perceived motor competence | 2.62±0.55 | 2.63±0.57 | 2.61±0.53 |
| PA enjoyment | 4.38±0.80 | 4.41±0.79 | 4.34±0.81 |
| Wellbeing | 2.61±0.41 | 2.59±0.40 | 2.63±0.42 |
| **Family-level correlates** |  |  |  |
| Caregiver age (years) | 37.30±4.61 | 37.27±4.73 | 37.34±4.48 |
| Caregiver BMI (kg/m^2^) | 24.01±5.15 | 24.31±5.47 | 23.68±4.75 |
| Caregiver LPA (mins/day) | 16.71±16.94 | 16.65±18.34 | 16.78±15.23 |
| Caregiver MVPA (mins/day) | 27.23±20.92 | 30.30±23.95 | 23.80±16.25 |
| Number of children in family |  |  |  |
| 1 | 398 (39.3%) | 247 (46.2%) | 151 (31.7%) |
| 2 | 539 (53.3%) | 257 (48.0%) | 282 (59.1%) |
| ≥3 | 75 (7.4%) | 31 (5.8%) | 44 (9.2%) |
| Father education level |  |  |  |
| Below college | 705 (69.7%) | 379 (70.8%) | 326 (68.3%) |
| College or above | 307 (30.3%) | 156(29.2%) | 151 (31.7%) |
| Mother education level |  |  |  |
| Below college | 618 (61.6%) | 330 (61.7%) | 288 (60.4%) |
| College or above | 394 (38.9%) | 205 (38.3%) | 189 (39.6%) |
| Monthly household income (yuan) | |  |  |
| Low income | 496 (49.0%) | 268 (50.1%) | 228 (47.8%) |
| Middle income | 405 (40.0%) | 218 (40.7%) | 187 (39.2%) |
| High income | 111 (11.0%) | 49 (9.2%) | 62 (13.0%) |
| Parental support for children’s PA | 2.60±0.82 | 2.65±0.82 | 2.54±0.81 |
| **Environment-level correlate** |  |  |  |
| Family PA environment | 1.76±0.54 | 1.84±0.58 | 1.66±0.48 |

Note：LPA=light physical activity, MVPA= moderate and vigorous physical activity, SB=sedentary behaviors, SLP=sleep duration, BMI=body mass index. Data are presented as Mean±SD or n (%).
